# Machine-learning improves understanding of glass formation in metallic systems†

**DOI:** 10.1039/d2dd00026a

**Published:** 2022-06-14

**Authors:** Robert M. Forrest, A. Lindsay Greer

**Affiliations:** Department of Materials Science and Metallurgy, University of Cambridge UK rmf48@cam.ac.uk

## Abstract

Glass-forming ability (GFA) in metallic systems remains a little-understood property. Experimental work on bulk metallic glasses (BMGs) is guided by many empirical criteria, which are often of limited predictive value. This work uses machine-learning both to produce predictive models for the GFA of alloy compositions, and to reveal insights useful for furthering theoretical understanding of GFA. Our machine-learning models apply a novel neural-network architecture to predict simultaneously the liquidus temperature, glass-transition temperature, crystallization-onset temperature, maximum glassy casting diameter, and probability of glass formation, for any given alloy. Feature permutation is used to identify the features of importance in the black-box neural network, recovering Inoue's empirical rules, and highlighting the effect of discontinuous Wigner–Seitz boundary electron-densities on atomic radii. With certain combinations of elements, atomic radii of different species contract and expand to balance electron-density discontinuities such that the overall difference in atomic radii increases, improving GFA. We calculate adjusted radii *via* the Thomas–Fermi model and use this insight to propose promising novel glass-forming alloy systems.

## Abstract 2

We train a neural-network model for glass formation in metallic systems, and probe its inner workings to extract theoretical insights.

## Graphical abstract

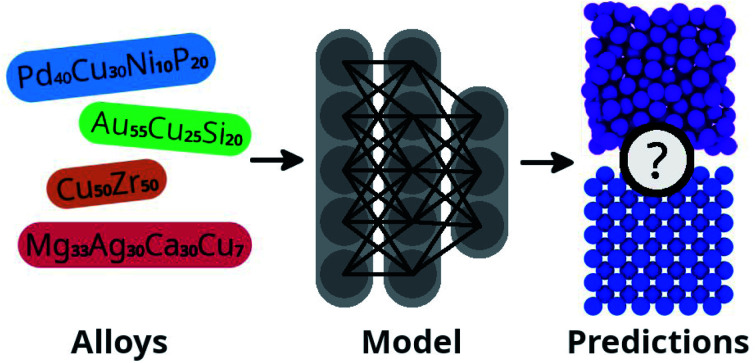

## 1 Introduction

*Metallic glasses* (MGs) are created from molten metal alloys by cooling at a rate fast enough to prevent any significant degree of crystallization.^1^ The lack of time for transition into a crystalline ordering leaves the atoms in a liquid-like structure.^2^ Alloy compositions for which it is possible to produce samples with a minimum thickness of 1–10 millimetres are referred to as *bulk* metallic glasses (BMGs), while others may be obtainable only as thin glassy ribbons (GRs).^3,4^

The amorphous structures of MGs give them interesting properties, with potential applications in many areas, from sporting equipment to aircraft and automotive components.^5–9^ The relative novelty of MGs means further glass-forming alloy compositions are awaiting discovery, with the promise of meeting previously inaccessible requirements for applications.

The ability of an alloy composition to resist arrangement into an ordered crystalline phase upon cooling is referred to as the *glass-forming ability* (GFA). The GFA of an alloy composition may be directly quantified *via* the maximum achievable diameter of a rod cast into a fully glassy state, *D*_max_.^10^

Predicting the glass-forming ability of alloy compositions is a central goal in MG research.^11^ This research is plagued by empirical rules and trial-and-error experimentation, with frustratingly many proposed criteria for GFA,^4^ each often published with claims of superiority yet limited proven applicability. The present work explores the power of the burgeoning ‘fourth paradigm’ of scientific discovery, that being the utilization of data to train machine-learning models of physical phenomena, which in turn inform our theoretical understanding.^12^

Machine-learning (ML) involves the creation of models that can improve their performance *via* exposure to data.^13^ Materials science is no exception to the rapid spread of ML as a research tool. ML approaches to materials science use the vast amount of experimental data now available to model the physical laws governing observed phenomena.

Neural networks, in particular, are commonly applied in ML, and have been widely used within materials science to model atomic interactions,^14^ predict synthesis routes,^15^ reconstruct structures from imaging,^16^ identify phases and transitions,^17^ predict welding criteria,^18^ predict material properties,^19^ and to predict the existence of novel materials,^20^ among others.^21,22^

In recent years there has been interest in applying ML to produce predictive models for the GFA of alloy compositions.^23–27^ Such models are increasingly a key tool for the discovery and optimization of novel glass-forming alloy compositions, enabling researchers to reduce the amount of expensive and time-consuming trial-and-error experimentation which, despite empirical guides such as Inoue's rules,^28^ is required given the lack of understanding of glass formation. Several recent studies^10,29,30^ have demonstrated the capability of screening driven by ML models to identify new glassy-alloy candidates.

In this work, we produce a neural-network model that simultaneously addresses the thermodynamic and kinetic factors influencing glass formation. Other published models usually focus on a single aspect, as is seen with the separate models for the glass transition and crystallization onset temperatures of Jeon *et al.*,^31^ the models for *D*_max_ of Peng *et al.*^32^ and Reddy *et al.*,^33^ and the GFA classifiers of Liu *et al.*^34^ and Sun *et al.*^35^ The novel architecture we present in the present work, described in detail in Section 2, gives a single neural-network model able to produce simultaneous predictions of multiple alloy properties, that exploit mutually beneficial shared learning. We train this model using a wide variety of data known to be associated with glass formation, including atomic radii,^36^ valence electron concentration,^37^ mixing enthalpy,^28^ and mismatch entropy.^38^

Furthermore, we seek to go beyond training a predictive model for GFA. Our central goal is to obtain insights into the mechanisms of glass formation by analysis of the inner-workings of the model. Our use of physical data as input to the model, rather than simply the atomic percentages of elements in alloy compositions as seen in other works,^31^ enables the investigation of specific properties and their relation to GFA. Similar work has been performed by Dasgupta *et al.*^39^ who gained insight into the relations between deep eutectics and GFA *via* ML analysis of phase diagrams, and by Kaufmann *et al.*^40^ who identified key chemical factors for the design of high-entropy ceramics from analysis of a random-forest model. Furthering our fundamental understanding of physical processes in this manner brings benefits which transcend the predictive power of any particular ML model, reinforcing our theoretical foundations and reducing the need to rely on empiricism.

## 2 Neural-network architecture

The open-source machine-learning framework *Tensorflow*^41^ is used here to construct neural networks tasked with predicting the following properties for an alloy composition:

• The liquidus temperature *T*_l_,

• The temperature of the onset of crystallization *T*_x_,

• The glass-transition temperature *T*_g_,

• Classification of GFA as crystalline, GR, or BMG,

• The maximum casting diameter of a fully glassy rod *D*_max_.

**Fig. 1:**
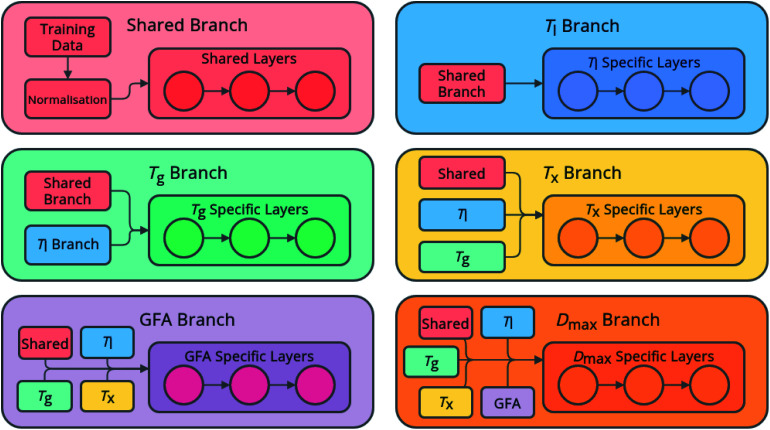
Architecture of the neural-network models, showing globally accessed shared branches, and sequentially assembled prediction branches for the liquidus temperature, *T*_l_, the glass-transition temperature, *T*_g_, the temperature of crystallization onset, *T*_x_, glass-forming ability (GFA) classification as crystal, glassy ribbon, or bulk metallic glass, and the maximum castable diameter of a fully glassy rod, *D*_max_.

The networks consist of a number of densely linked layers of neurons leading to multiple output neurons (Fig. 1), allowing one model to be trained to predict all of the target properties. The use of shared layers allows the models to learn globally useful features, before specializing in specific branches for each feature. Further, predictions for each feature are fed sequentially as input for other features, in the order listed above, meaning that relationships between the predicted features can also be exploited. This approach is novel both in the sequential flow of predictions to inputs through the model, and in its ability to simultaneously predict multiple properties of an alloy composition without the requirement to train multiple individual models.

All features enter the network through a normalization layer, in which they are scaled to have zero mean and unit variance. The ReLU activation function is used by all hidden layers. Predictions are obtained *via* a *softmax*^42^ activated neuron for classifications,1
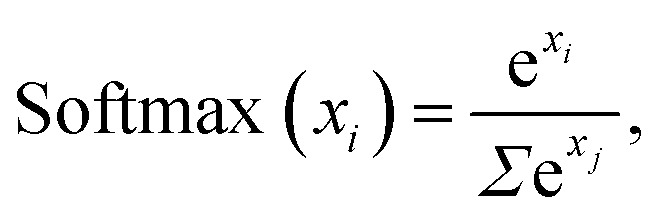
and a *softplus*^43^ activated neuron for regressions,

2 Softplus (*x*) = log(e^*x*^ + 1).

Softplus is used to ensure positive-valued predictions, as none of the regression targets can exhibit negative values.

*Dropout* layers, which eliminate a percentage of inputs to avoid overfitting,^44^ are inserted before each hidden layer with a dropout rate of 30%. *Regularization*, a technique to penalize model complexity during training and to deter overfitting,^45^ is applied to all layers using the L2-norm form with a rate of 0.001. Finally, the *max-norm* constraint^46^ is applied to all hidden layers, ensuring the maximum magnitude of the weights does not exceed a value of 3, further reinforcing the favourability of simple models.

Hyperband hyperparameter tuning^47^ is used to identify the optimal number of layers and number of neurons per layer for the network. Some manual intervention is performed to select parameters with the best performance while remaining computationally feasible with the available resources. The hyperparameters used are listed in Table 1.

**Table 1:** Hyperparameter values of the neural-network model, as determined by hyperband tuning

Hyperparameter	Value
Number of shared layers	3
Number of specific layers	5
Nodes per layer	64

The training process for a neural network involves minimizing the *loss*, which is a measure of how well the outputs of the model match the true values, with lower values indicating a better fit.^48^ Here, the loss is calculated for the regression predictions using the *Huber loss* function,^49^3
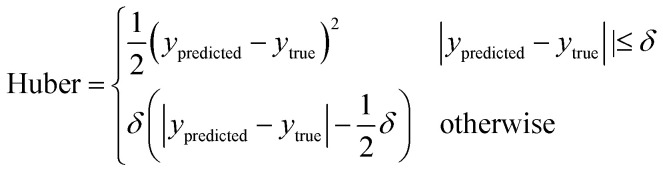
where the parameter *δ*, here set to 1.0, controls the transition between linear and parabolic loss, the former being more lenient on errors than the latter.

For classification of GFA, the *categorical cross-entropy* (CE) is used,^50^4
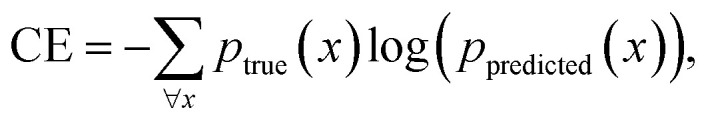
where *x* is a training example, *p*_true_ is the true probability distribution, returning 100% for the true class of *x*, and *p*_predicted_ is the predicted distribution that describes the confidence of the model regarding the class of *x*.

The Huber loss function is used here rather than more common loss functions, such as the mean-squared-error (MSE), as it is more robust in the presence of outliers, something of particular importance because of the unbalanced nature of the *D*_max_ training data.

The multi-output nature of the models requires the individual losses for each prediction to be summed to obtain the overall loss, which is the quantity minimized during training. Since the numerical scales involved differ between each of the predictions, the value of the loss for temperature predictions may be in the range 10^2^ to 10^3^, while for GFA and *D*_max_ this may be 10^0^ to 10^1^. To ensure that optimization of the loss function does not heavily favour reducing the loss associated with the temperature predictions, the individual loss functions are weighted before summation. The weights scale the natural ranges of each loss component to the same approximate order of magnitude. The weights are also tuned to favour reduction of the GFA and *D*_max_ losses, as these are the principal quantities of interest in this investigation.

The Adam optimizer^51^ is used to perform minimization of the loss function, with an initial learning rate, a parameter that controls the size of the steps taken during minimization, of 0.01.

Further detail on neural-network models is provided in ESI S1.†

## 3 Data

**Fig. 2:**
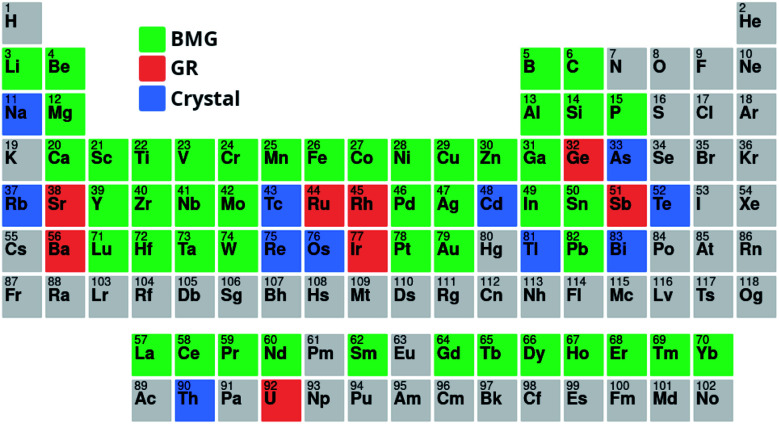
Periodic table colour-coded per element by the highest glass-forming ability, from bulk metallic glass (BMG) to glassy ribbon (GR) to crystal, of any alloy in the dataset containing that element. Elements without any colour-coding were not present in any alloys in the dataset.

The dataset used here to train neural networks is a compilation of datasets used previously in GFA modelling works,^27,29^ and data on pure elements. The dataset includes the following experimentally measured properties for each alloy: *T*_l_, *T*_x_, *T*_g_, *D*_max_, and classification as either a crystal, GR, or BMG. Included in the dataset are 1700 (25.6%) crystals, 3763 (56.7%) GRs, and 1175 (17.7%) BMGs. Fig. 2 illustrates the range of elements for which data are available. The precedent of approximating the *D*_max_ of GRs to 0.15 mm is followed.^27^

To counteract the lack of balanced class representation, the relative importances of samples are weighted in inverse proportion to the percentage of their class. This ensures that the model does not overly focus learning on the most populous class, where the biggest potential decrease in the loss function is available. It is noted that an experimental measurement of crystallinity does not preclude the future observation of, given the right experimental circumstances, an alloy forming a GR or BMG. Similarly, measurement of a composition forming a GR does not prevent that same composition later being observed to form a BMG. Due to these inherent uncertainties, it is expected that classification models trained on the experimental data will not be able to attain high accuracy, as some compositions may be recorded as crystalline because of experimental limitations rather than their inherent GFA. The challenge of training a model for *D*_max_ is compounded by issues with reported experimental measurements. *D*_max_ is defined as the maximum castable diameter of a fully glassy rod, and depends significantly on how the sample is fabricated.^52^ Precision in reported measurements of *D*_max_ is usually low, extending only to a tenth of a millimetre, and often there is rounding to numbers such as multiples of 5 or 10 mm.

**Fig. 3:**
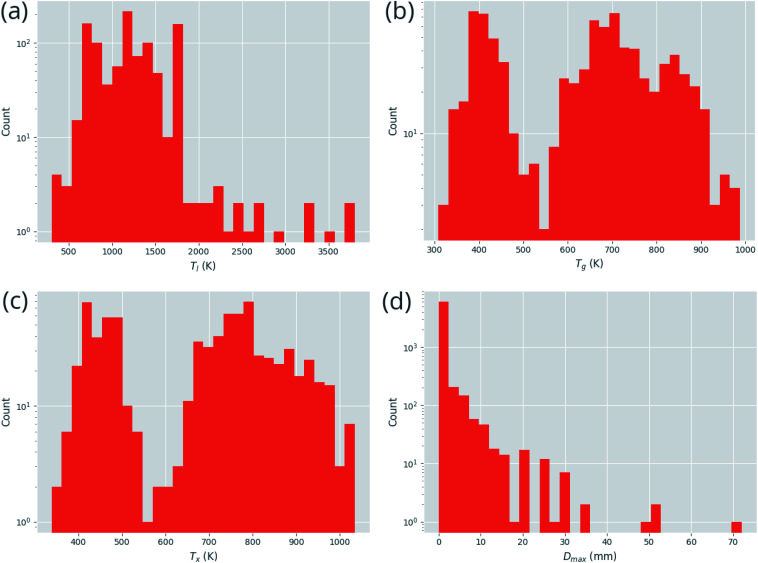
Distributions of continuous prediction targets in the dataset, (a) the liquidus temperature, *T*_l_, (b) the glass-transition temperature, *T*_g_, (c) the temperature of crystallization onset, *T*_x_, and (d) the maximum castable diameter of a fully glassy rod, *D*_max_.

Fig. 3 demonstrates the distribution of the numerical target features in the dataset. While *T*_l_, *T*_x_, and *T*_g_ are fairly well represented across the range of values, *D*_max_ exhibits a clear imbalance in its distribution, with far more small values than large. Creating a model for such an unbalanced distribution is a significant challenge for machine-learning, as the general rules governing the underlying processes are not fully demonstrated.

For each composition in the dataset, features are calculated based on the properties of the constituent elements. These features serve as the “inputs” used by the model to identify relations to the target data.

Simple properties are calculated using the linear-mixture rule,5
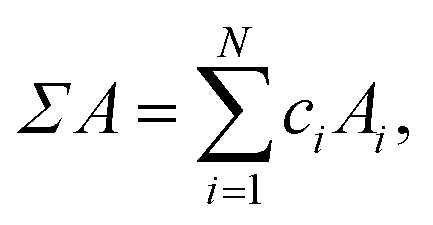
where *c*_*i*_ is the atomic percentage of the composition consisting of element *i*, and *A*_*i*_ is the value of property *A* of pure element *i*. The deviation between elemental properties within compositions is also considered,^53^6
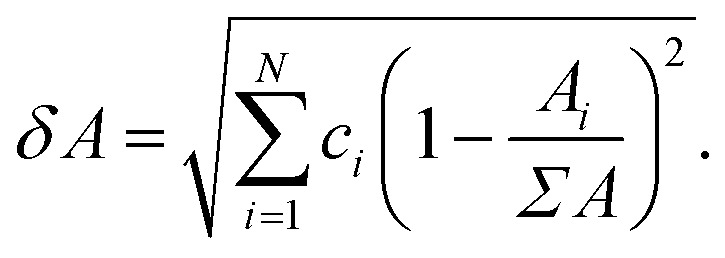


*Σ* and *δ* prefixes are used throughout to denote the linear mixture and deviation of a property, respectively. Table 2 lists all of the features used during training. The set of used features is obtained following culling of highly correlated and static features from a larger set of candidate features, detailed in Tables S1 and S2 (in ESI†). Some features demonstrate little variation throughout the entire dataset: these are unlikely to present any particularly useful information to the model, as they cannot be used to distinguish one alloy composition from another. The variability of features is calculated using the quartile coefficient of dispersion (QCD),^54^7
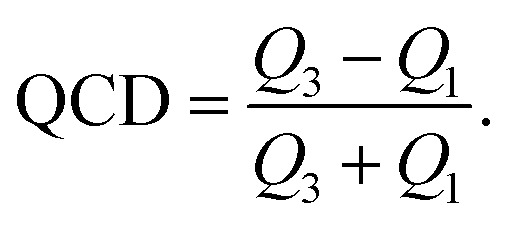


**Table 2:** Calculated features remaining after culling those that are of high correlation (*p* > 0.8) or low variation (QCD < 0.1). *Σ* & *δ* refer to the linear mixture and discrepancy respectively, of a feature. These features form the input data used by the models to make predictions for alloy compositions

*Σ* & *δ* universal sequence number^55^	*Σ* & *δ* Debye temperature	*Σ* & *δ* fusion enthalpy
*Σ* & *δ* valence	*Σ* & *δ* valence electrons	*Σ* & *δ* electron affinity
*Σ* & *δ* thermal conductivity	*Σ* & *δ* thermal expansion	*Σ* & *δ* group
*Σ* vaporization enthalpy	*Σ* period	*Σ* modified Mendeleev number^56^
*Σ* shell to Mendeleev number ratio	*δ* series	*δ* crystal structure
*δ* density (s.t.p.)	*δ* Pettifor Mendeleev number^57^	*δ* neutrons
*δ* cohesive energy	*δ* melting temperature	*δ* chemical hardness
*δ* chemical potential	*δ* radius	*δ* Wigner–Seitz boundary electron-density^58^
Mixing Gibbs free energy	Mixing entropy	Viscosity^27,59^
Lattice distortion^60^	p-Valence proportion	Mixing *P*_HSS_^61^

Any feature with a QCD below a threshold of 0.1 is removed from the dataset. Some features in the dataset are highly correlated; they thus present approximately the same information to a model. Therefore, only one member of a correlated group of features is required; the others, being redundant, act only to slow down the training process.

**Fig. 4:**
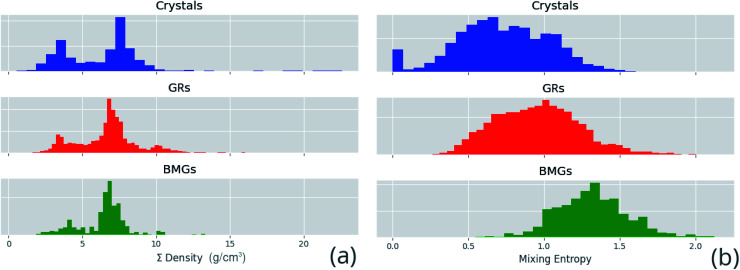
Distributions of a sample of calculated features for each alloy composition in the dataset, (a) the linear mixture of density and (b) the mixing entropy, separated by GFA classification of crystal, glassy ribbon (GR) or bulk metallic glass (BMG).

**Fig. 5:**
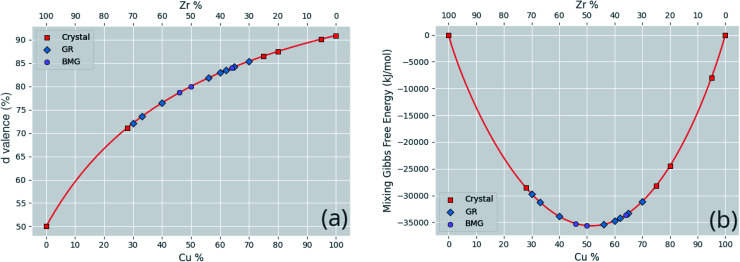
Variations of a sample of calculated features (a) the d-valence proportion and (b) the Gibbs free energy of mixing, across the Cu–Zr alloy composition. Specific compositions found in the dataset are labelled corresponding to their GFA classification of crystal, glassy ribbon (GR), or bulk metallic glass (BMG).

Correlated features are culled from the dataset following the technique of Liu *et al.*;^34^ for each pair of highly correlated features (*p* > 0.8), the feature with the lower variance throughout the dataset, here measured by the QCD, is discarded. A number of corrections are made to the datasets sourced from the literature before they are used in this work, including correcting atomic percentages in alloy compositions, normalizing notation such that all percentages sum to 100 (as some instead summed to 1), and correcting instances of experimental data being entered into the wrong columns. Where duplicate entries occur, for example two or more experimental investigations of the same alloy composition, their results are averaged. Fig. 4 shows the distributions of two of these properties across the dataset, and Fig. 5 demonstrates the variation of some properties across a single composition-space, showing that some features have an immediately noticeable relationship with glass formation. The Gibbs free energy of mixing exhibits with lower values a trend towards higher GFA. This is, however, no immediate solution to glass formation, as the exact relation remains non-trivial. Conversely, the d-valence feature shows no correlation, in either its linear mixture or deviation, with GFA. The application of machine-learning to the problem of glass formation, while useful for creating predictive models, also provides a method for probing these complex interactions between atomic and chemical properties and GFA, as discussed in Section 5.

## 4 Training models

### 4.1 *k*-Folds cross-validation

**Fig. 6:**
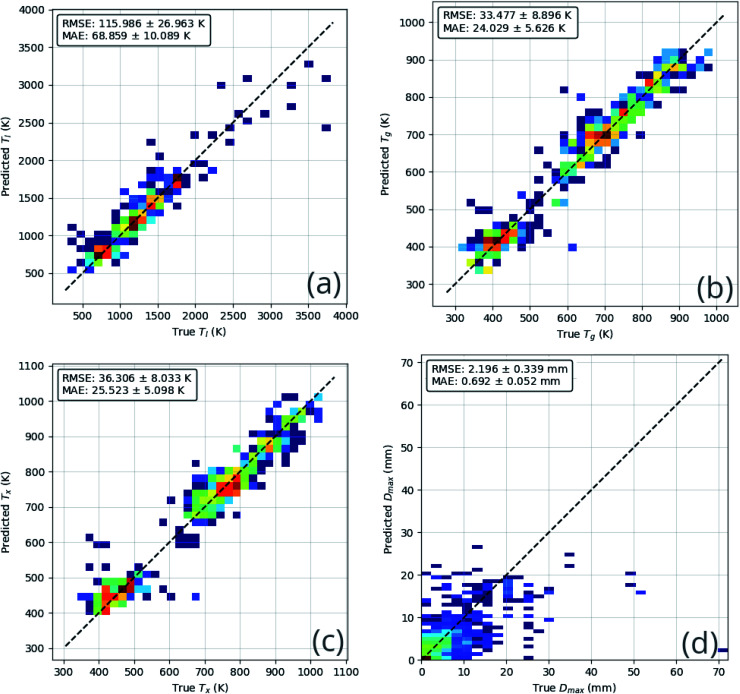
Heatmaps of neural-network model performance on testing data across the *k*-folds sets, for each of the regression targets: (a) the liquidus temperature, *T*_l_; (b) the glass-transition temperature, *T*_g_; (c) the temperature of crystallization onset, *T*_x_; and (d) the maximum castable diameter of a fully glassy rod, *D*_max_. Heatmaps aid visualization of the density of data around the ideal line of truth = prediction. Inset in each are the root-mean-square-error (RMSE) and mean-absolute-error (MAE) metrics, measuring the performance of the model on that target.

The technique of *k*-folds cross-validation involves splitting the dataset into *k* pairs of *training* and *test* subsets, such that each composition in the dataset is used for training (*k* − 1) times and for testing once.^42^ Here, we use the commonly chosen value of *k* = 5. Splitting of the dataset follows a similar approach to that taken by Ward *et al.*;^10^ rather than randomly assigning each composition to a subset, alloy groups are assigned together. This prevents the model from exploiting learning on a composition in the training subset when being evaluated on a very similar composition in the test subset, thus providing a more robust measure of general performance. The average performance on the test sets indicates the level to which the model has learned generalized rules, rather than overfitting and simply learning to replicate the training data. Fig. 6 and Table 3 show the performance of the model on each of the test sets. *F*_1_ scores, formally defined in ESI S1,† are provided in Table 3 as they evaluate the performance of the model while accounting for imbalance in the distribution of GFA classes in the dataset.

**Table 3:** Evaluation metrics on testing data across the *k*-folds, measuring the general ability of the neural-network model in predicting GFA-classifications of either crystal, glassy ribbon, or bulk metallic glass, and *D*_max_ values

Fold number	GFA accuracy (%)	GFA *F*_1_ score (%)	*D*_max_ RMSE (mm)
1	73.4	73.3	1.80
2	68.3	66.2	2.42
3	69.8	69.6	2.73
4	63.7	64.7	1.92
5	66.4	68.2	2.10
Average	68.1	68.4	2.20
Standard deviation	3.25	2.94	0.34

The results of the *k*-folds cross-validation process show that the model has the capacity to learn generally useful relations between the supplied feature information and the prediction targets, out-performing random guessing. For example, in the case of GFA classification with three possible classes, random guessing would return 33.3% accuracy.

### 4.2 Ensembling

The ensembling technique is applied to obtain more reliable and robust machine-learning models, wherein multiple ‘submodels’ are combined to produce predictions with the potential for better performance than any of the submodels alone.^62^

Since each submodel is trained on a different subset of the training data during the *k*-folds process, different associations between inputs and predictions are likely to be learned, and thus the submodels make different errors. Ensembling allows the combined model to exploit the strengths of each submodel, while avoiding the tendency of a single model to overfit to training data. Further, due to the nature of the learning algorithm, the training of a neural network is a highly stochastic process, resulting in some models by chance being better or worse than others. Ensembling multiple models acts to reduce this variance.

**Fig. 7:**
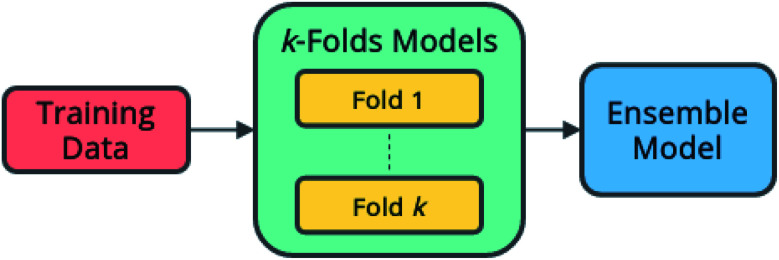
Ensemble model architecture. A secondary model learns to combine the predictions of multiple primary models.

Here, an ensemble model is created using submodels trained during *k*-folds cross-validation according to the architecture shown in Fig. 7. The predictions of each of the submodels become the input to a secondary model, which learns the optimal combination of submodels to produce the best predictions.

**Fig. 8:**
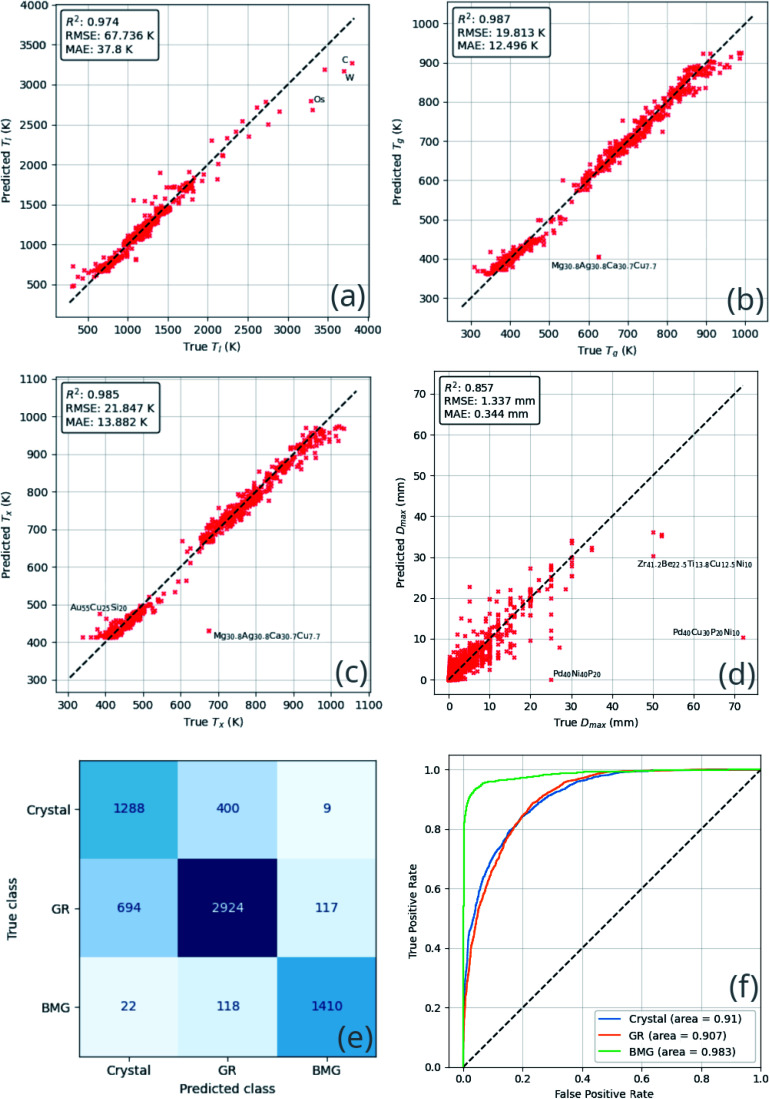
Comparison of ensemble neural-network model predictions and true values for each predicted quantity, (a) the liquidus temperature, *T*_l_; (b) the glass-transition temperature, *T*_g_; (c) the temperature of crystallization onset, *T*_x_; (d) the maximum castable diameter of a fully glassy rod, *D*_max_; (e) glass-forming ability (GFA) classification confusion matrix; and (f) the GFA receiver-operating characteristic. These comparisons demonstrate that the model is able to learn generally applicable rules that out-perform random guessing.

The performance of the ensemble model is illustrated in Fig. 8. The mean-absolute-error of each of the regression predictions is below the mean-absolute-deviation of the dataset itself, showing that the model is able to learn useful relations that out-perform random guessing.

Similar results are observed with the classification ability of the model. The confusion matrix and receiver-operating characteristic (ROC) curve^63^ show excellent ability to distinguish BMGs from GRs and crystals, and good but lesser ability to distinguish GRs from crystals. The overlap between the crystal and GR classes may imply that many of the crystal-forming compositions present in the training dataset could form GRs under different experimental circumstances.

Table 4 provides further classification metrics, each defined in ESI S3,† supporting the above evaluation of the model's ability.

**Table 4:** Classification metrics evaluating the ability of the ensemble model's GFA predictions overall, and for each category of glass-forming ability, showing excellent ability to identify bulk metallic glasses (BMGs), and good ability to distinguish crystals and glassy ribbons (GRs)

Metric (%)	Overall	Crystals	GRs	BMGs
Accuracy	80.5	83.9	81.0	96.2
Recall	81.7	75.9	78.3	91.0
Precision	80.3	64.3	85.0	91.8
Specificity	89.4	86.5	84.0	97.7
*F*_1_ score	80.8	69.6	81.5	91.4
Informedness	71.1	62.4	62.3	88.6
Markedness	69.1	56.1	62.0	89.2
Matthews correlation	70.1	59.1	62.2	88.9

A wide variety of performance metrics are provided for the classifier such that it is evaluated from a range of perspectives. No one metric fully describes the confusion matrix, and some may be actively misleading if considered in isolation. For example the accuracy of a classifier which always predicts ‘positive’ for a training set of 900 ‘positives’ and 100 ‘negatives’ is 90%. This high accuracy value may mistakenly be interpreted to mean the classifier is strong, despite no meaningful learning having taken place.

The ensemble model makes better predictions for all targets when compared to the *k*-folds models, and the most significant improvements are seen for *D*_max_ and GFA predictions. The ensemble model demonstrates a reduction in *D*_max_ RMSE of 0.859 mm (39.1%) and an improvement in overall GFA classification accuracy of 12.2% when compared to the average submodel. These results suggest that the ensemble model can be trusted to produce meaningful output when applied sensibly. However, as with any machine-learning model, as the area of application is moved further away from the bounds of the training data, confidence may decrease.

Outliers in terms of error in prediction of *D*_max_ include the 72 mm Pd_40_Cu_30_Ni_10_P_20_ BMG composition, and the similar Pd_40_Ni_40_P_20_ composition. While the training dataset contains many examples of Pd-containing compositions, the vast majority are GR-forming rather than BMG-forming, and few if any BMGs exhibit such large *D*_max_ values as 72 mm. This serves to highlight the difficulty in replicating extraordinary results using machine-learning or other statistics-based methods; the glimpses that rare examples provide of the general laws governing the processes being modelled may not be enough to reliably explain their occurrence.

#### 4.2.1 Comparison with other models

Table 5 compares the *D*_max_ prediction performance of the ensemble model to other models. This demonstrates that the novel approach used here is competitive with other more common methods found in the literature.^10,25–27,30^

**Table 5:** Comparison of the *D*_max_ prediction performance for the ensemble neural-network model of this work with various other published models. Multiple metrics are provided as each cited model was published with different analysis

Model type	*R* ^2^	MAE (mm)	NMAE (%)	RMSE (mm)	NRMSE (%)	Authors
Ensemble NN	0.86	0.34	0.48	1.34	1.86	*This work*
Random forest	0.89	0.21	0.29			Ward *et al.*^10^
Random forest	0.64					Deng *et al.*^25^
Random forest	0.85			1.20	2.40	Xiong *et al.*^26^
Random forest	0.77			2.89	8.26	Xiong *et al.*^26^
Symbolic regression	0.67			3.37	9.62	Xiong *et al.*^26^
Correlation NN	0.96			0.62	0.86	Samavatian *et al.*^27^
Levenberg–Marquardt NN	0.79			0.75	1.04	Zhou *et al.*^30^
Gaussian process regression	0.80			0.73	1.01	Zhou *et al.*^30^
Support vector regression	0.71			0.85	1.18	Zhou *et al.*^30^
Random forest	0.64			0.88	1.22	Zhou *et al.*^30^

In this comparison, the cited publications did not all test their models across the same range of *D*_max_ values, with some culling outliers such as the 72 mm Pd_40_Cu_30_Ni_10_P_20_ BMG to improve training on the larger population of BMGs with lower *D*_max_. As a result, the published values of the mean-absolute-error (MAE) and root-mean-square-error (RMSE) cannot be directly compared either to this work, or amongst themselves. To enable direct comparison with the literature models, the normalized mean-absolute-error (NMAE) and normalized root-mean-square-error (NRMSE) are calculated by division of the published values by the range of *D*_max_ considered during their evaluation.^64^

The multi-output nature of the ensemble model created in this work allows for a simplification of predictive workflows used when investigating multiple trial alloy properties. Only a single model is required to be queried, rather than a collection of different models, which may have differing interfaces.

## 5 Guiding understanding

Currently, the composition dependence of the glass-forming ability of metallic systems is understood largely empirically. As such, the importance of specific information to successful machine-learning models is of high interest. Features with large influence may highlight the specific physical properties or processes that define GFA, leading to better understanding.

Neural-network models are often referred to as ‘black boxes’ due to their internal reasoning not being directly visible to humans.^65^ As such, the indirect method of *permutation importance*^66^ is applied here to probe the reliance of the models on individual features. Each feature is, in turn, reassigned from the original alloy composition for which it was calculated to another random alloy composition. This destroys the relation of the feature to the rest of the dataset, and its composition-dependence. The model, with the modified dataset, is then evaluated using the *k*-folds cross-validation method; significant degradation of performance relative to the model with the original dataset signals that the permuted feature is a key indicator for the properties being predicted.

**Fig. 9:**
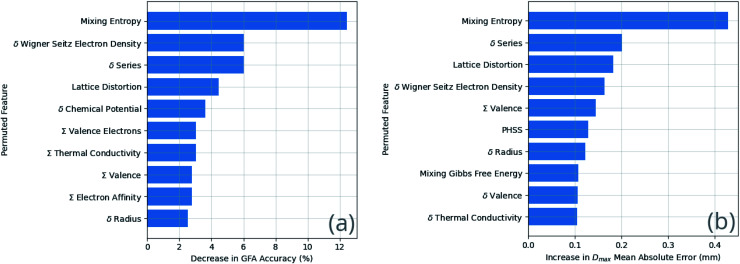
The top ten most important features as determined by feature permutation applied to the ensemble neural-network model for (a) GFA classification and (b) prediction of the maximum castable diameter of a fully glassy rod, *D*_max_. The most important features cause the largest decrease in model performance when permutation destroys their relation to the other data.

The results of the feature permutation tests shown in Fig. 9 suggest recovery of Inoue's rules, and of the confusion principle. Inoue's first rule and the confusion principle are both identified *via* the importance of the mixing entropy, and Inoue's second rule *via* the atomic-radius deviation.

There is an element of self-fulfilling prophecy to consider, depending on the rate of publishing of null results by those searching for glass-forming compositions. If the searches are being driven by criteria such as Inoue's rules, and only the positive results are published and appear in a dataset such as that used in this work, then the effectiveness of those criteria is artificially boosted due to a lack of counter-examples. It is of vital importance for machine-learning work that failures in the search for BMG-forming alloy compositions are published as well as successes, allowing ML to learn to distinguish between them. Some of the features measured to be important are likely due to sampling bias rather than physical causation. For example, smaller ranges of certain features may be represented in BMGs when compared to crystals and GRs due to the relatively small sample of BMGs in the dataset. Further, the identification of certain features to be important is not immediately of significant assistance. An example is the deviation in series, since the series of an element, for instance iron being a transition metal, is a proxy for a variety of other properties.

**Fig. 10:**
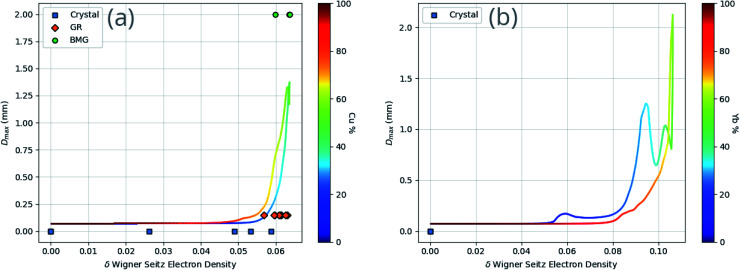
Dependence of *D*_max_ predictions on the Wigner–Seitz boundary electron-density deviation (*δn*_WS_) for two example binary compositions, (a) Cu–Zr and (b) Yb–Mn, showing an apparent association between high *δn*_WS_ and large *D*_max_. Specific compositions found in the dataset are labelled corresponding to their GFA classification of either crystal, glassy ribbon (GR) or bulk metallic glass (BMG). The colour bar enables reading of the atomic percentages of the binary composition.

While the identification of particular features as important is useful, it does not reveal the specifics of the relationship between the features and GFA. Fig. 10 demonstrates the dependence of GFA and *D*_max_ predictions on the Wigner–Seitz boundary electron-density deviation for two alloy compositions, showing that larger deviation appears to be associated with higher predicted GFA in these cases.

The Wigner–Seitz boundary electron-density, *n*_WS_, is a key parameter in the Miedema model definition of the mixing enthalpy.^58^ If the value of *n*_WS_ for two pure elements is different, this is taken into account upon alloying by adjustment of the electronic structure such that there is no discontinuity, by compression of the Wigner–Seitz cell with lower *n*_WS_ and expansion of the cell with higher *n*_WS_. Any such transformation away from the equilibrium energy minimum of the pure substances must involve a positive contribution to the enthalpy of mixing. If the electronic structures of the alloying elements are sufficiently incompatible, a mixed solid solution may be energetically undesirable, or unstable if able to form. This information is captured by the Miedema model definition of the mixing enthalpy of two elements A and B,^67^8
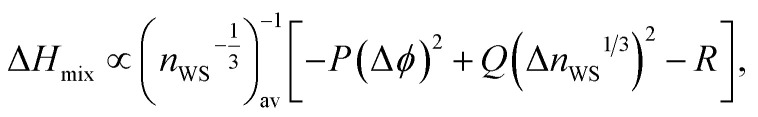
where 
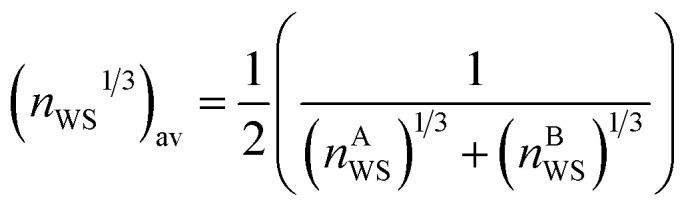
, 
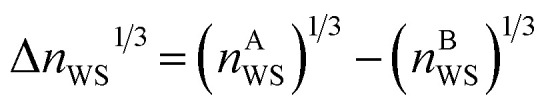
, Δ*Φ* is the difference in electronegativity, and *P*, *Q*, and *R* are empirical constants related to the periodic-table series of the elements A and B.

A larger difference in *n*_WS_ between mixed elements results in a more positive value of the mixing enthalpy, but this contribution may be outweighed by the negative contribution of the difference in electronegativity. A larger difference in electronegativity between alloying elements indicates increased bonding compatibility due to sharing of electrons being more energetically favourable. The sign of the mixing enthalpy of an alloy composition thus depends on the relative magnitudes of the electron-density discontinuity and the difference in electronegativity.^68^

The Wigner–Seitz radius, *r*_WS_, is the radius of a sphere of the same volume as the Wigner–Seitz cell, and is related to the conventional atomic radius. Upon alloying, the compression and expansion of Wigner–Seitz cells thus is equivalent to changes in the effective atomic radii of the elements present; Table 6 presents several resizing scenarios for a binary composition. In scenarios where the element with larger radius also has the larger *n*_WS_, the resulting Wigner–Seitz cell adjustments lead to an increase in the difference between the effective radii. This can be generalized to alloy compositions of arbitrary elements by comparing the elemental values to the alloy's linear mixture of radii, and noting an increase or decrease in the deviation of radii. Further, a larger discontinuity in *n*_WS_ necessitates a larger change in the effective radii to compensate, amplifying the corresponding increase or decrease in deviation of radii.

**Table 6:** Impacts on the effective atomic radii and resulting deviation of radii (*δr*), of the initial radii and the Wigner–Seitz boundary electron-density discontinuity, in a binary AB alloy composition

Isolated radii difference	*n*_WS_ difference	Effect
*r*_a_ ≥ *r*_b_	*n*_a_ > *n*_b_	*r*_a_ increases, *r*_b_ decreases, *δr* increases
*r*_a_ ≥ *r*_b_	*n*_a_ < *n*_b_	*r*_a_ decreases, *r*_b_ increases, *δr* decreases
*r*_a_ ≤ *r*_b_	*n*_a_ > *n*_b_	*r*_a_ increases, *r*_b_ decreases, *δr* decreases
*r*_a_ ≤ *r*_b_	*n*_a_ < *n*_b_	*r*_a_ decreases, *r*_b_ increases, *δr* increases
Any	*n*_a_ = *n*_b_	No change

Referring to Inoue's rule of large difference in atomic radii, this suggests that alloy compositions with a higher GFA may be designed by selecting elements such that those with the largest radii also have the largest *n*_WS_. This must however be balanced by a sufficient difference in electronegativity in order to maintain a large negative value of the mixing enthalpy. This idea is reminiscent of earlier work by Turnbull,^69^ suggesting that high GFA may be found in alloys of transition metal elements (A) with metalloidal or electropositive elements (B). In these alloys, Turnbull proposes that the repulsive pair-potentials of A–A and B–B interactions have minima at larger atomic distances than the A–B interaction, due to the outer electrons of B spreading to occupy the space near A; this redistribution of electrons causes a change in effective atomic radius.

To further probe the hints provided by analysis of the neural-network model, calculations are performed of the impact on the atomic radii in an alloy composition of the deviation of *n*_WS_ values. The Thomas–Fermi model relates the electron density to the radial distance from the nuclear core as,^70^9
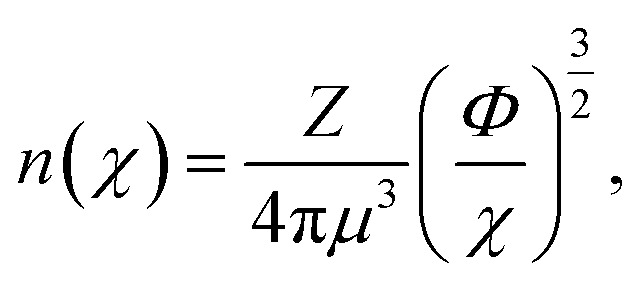
where *Z* is the atomic number, *μ* = *a*_0_(9π^2^/128*Z*)^1/3^, *χ* = *r*/*μ* is the dimensionless radius, and *Φ* is the Thomas–Fermi function which satisfies the Thomas–Fermi equation,10
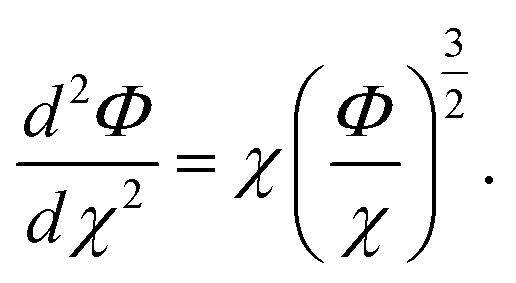


The Sommerfeld approximation^71^ provides a reasonable solution to eqn (10) when calculating electron densities away from the nucleus, and as such is applicable here for calculations at the Wigner–Seitz cell boundary. Sommerfeld's approximation defines *Φ* as,11
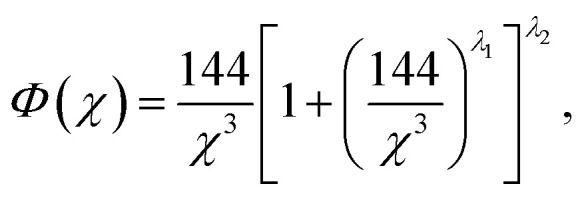
where *λ*_1_ = 0.2573 and *λ*_2_ = −3.886.

The electron density reached after balancing of the discontinuities between Wigner–Seitz cells depends on the cohesive energies of the elements involved, as those with greater cohesion resist changes to their atomic radius. In this work, we apply the empirical lever rule of Li,^72^ modified to account for different atomic percentages of each element in an alloy,12
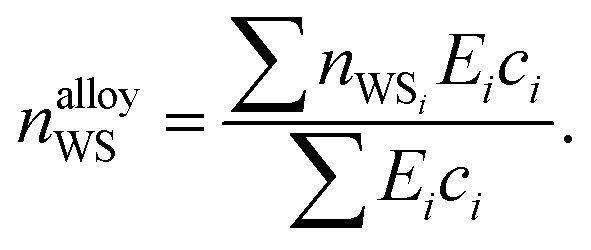


**Fig. 11:**
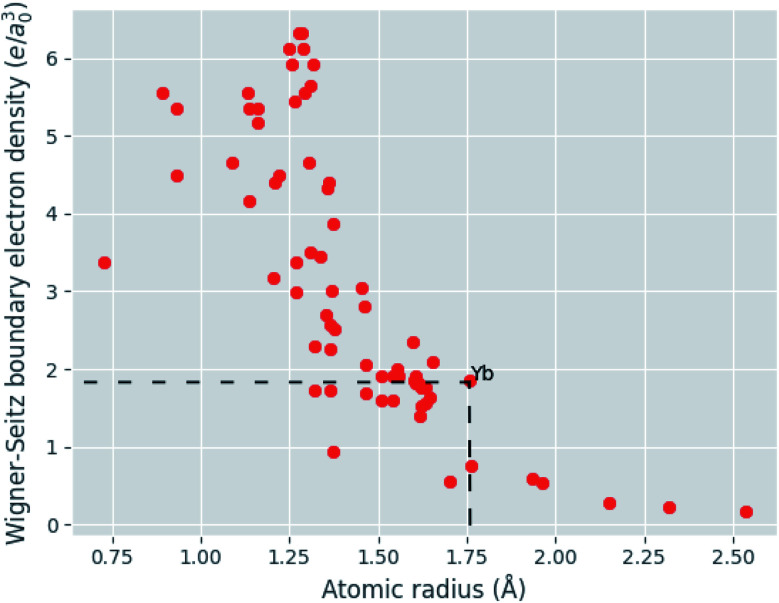
Illustration of the generally negative correlation between atomic radius and the electron density at the Wigner–Seitz cell boundary (*n*_WS_). In a binary composition, increases to the deviation in radii are possible when the largest atom also has the largest *n*_WS_. The pool of candidate elements able to exploit this effect to increase GFA is exemplified for Yb by dashed lines.

**Fig. 12:**
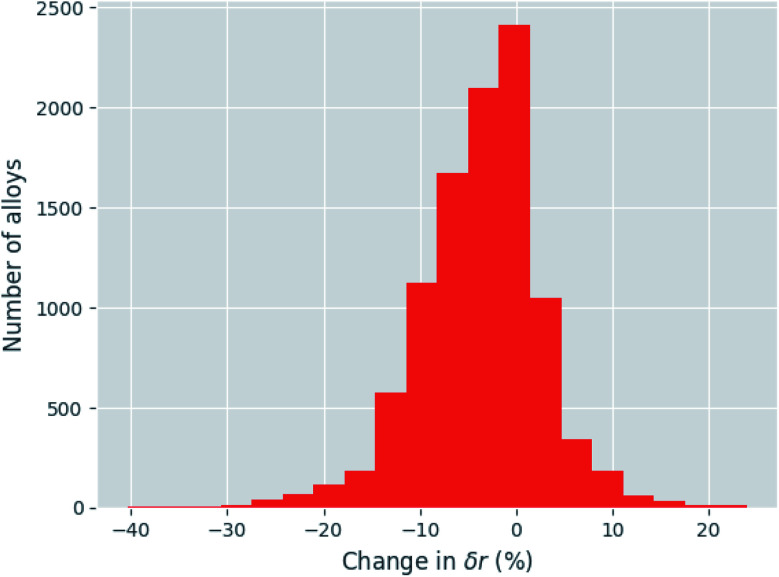
Histogram of the change in deviation in atomic radii (*δr*) caused by accounting for relaxation of discontinuities in electron density between Wigner–Seitz cells, calculated for a random sample of 10 000 binary to quaternary alloys, demonstrating that for a large majority of alloys *δr* decreases, corresponding to lower GFA under Inoue's empirical rules.

Given eqn (9), (11) and (12), the change in atomic radii of elements in any given alloy composition can be approximated. As mentioned previously, this change may either increase or decrease the deviation of atomic radii in the composition, influencing its GFA under Inoue's rules. While it is unlikely that the neural network is able to uncover this exact physics during training, it may discover some approximate relationship between GFA classification and *n*_WS_ as a proxy for modification of the deviation in radii. This concept is reversed when considering high-entropy alloys (HEAs), the formation of which is promoted by a small difference in radii.^73^ There are far more possible HEA-forming alloy combinations that can take advantage of the electron-density-driven balancing of radii than there are glass-formers, since the radius generally correlates negatively with *n*_WS_^74^ (Fig. 11). This point is further demonstrated in Fig. 12, in which the majority of a large sample of alloys exhibit decreased *δr* when accounting for changes in radii, meaning most alloys may have lower GFA than would be suggested by application of Inoue's rules using standard radii.

To suggest promising directions in the search for novel glass-forming alloys, we consider the 66 elements highlighted in Fig. 2. From these elements, it is possible to form 2, 145 binary alloys, and 45, 760 ternary alloys. This number of possible alloys continues to grow combinatorially as more elements are included. Applying constraints, we consider only the alloys with negative mixing enthalpy, such that a mixed solid solution can always be formed. We further limit consideration to those alloys with a deviation in radii which has increased after radii adjustments due to *n*_WS_ discontinuities, and is at least 12%, as per Inoue's rules.^28^ Then, 52 binary and 1, 279 ternary alloy systems match these criteria, approximately 2% of each group. Table 7 lists 10 binary and 10 ternary systems of interest, and the probability of glass-formation as predicted by the ensemble neural-network model. Lithium and sodium are common among these alloy systems, having among the lowest radius and *n*_WS_ combinations. The addition of such elements to existing glass-forming alloy compositions may be a possible method of increasing GFA, and indeed this has been observed with MgCuY alloys.^3^ Identified alloy systems already known to form glasses, such as the Mo–Rh–B system,^75^ are excluded from Table 7.

**Table 7:** Alloy systems identified to exhibit increased radii deviations after accounting for changes in radii due to *n*_WS_ discontinuities, and which have negative mixing enthalpies, accompanied by the probability of glass formation as predicted by the ensemble neural-network model

Alloy system	Probability of glass formation
Pd–Th–Na	98%
Au–Th–Na	97%
Pt–Zr–Na	95%
Pt–Nd–Na	93%
Nd–Re–C	90%
Fe–Pt–Li	87%
Pt–W–C	87%
Pd–Na	85%
Pt–Cu–Li	83%
Pt–Na	80%
Mn–Ru–C	76%
Au–Li	74%
Pt–Li	72%
Co–Ir–Li	72%
Nd–Te	67%
Ir–Nd	62%
W–C	58%
Pt–C	56%
Pr–Re	53%
Al–Li	25%

## 6 Conclusion

Understanding of glass formation in metallic systems lacks a comprehensive theoretical basis, which is not conducive to the development of new glass-forming alloys. To obtain insights into the nature of glass formation, we train a machine-learning (ML) model of novel ensemble neural-network architecture to predict the glass-forming ability (GFA) of alloy compositions, and apply the technique of feature permutation to probe its internal reasoning. We recover the well-known GFA criteria of Inoue's rules and the confusion principle, and are led to further consideration of the influence on GFA of changes in atomic radii upon alloying due to the balancing of discontinuities in the Wigner–Seitz boundary electron-density *n*_WS_. We find that with certain combinations of elements, wherein the atoms with larger atomic radius also have higher *n*_WS_, the difference in radii increases upon alloying as the larger atoms expand and the smaller atoms contract. We propose that high-GFA alloy compositions may be designed by selecting elements specifically to induce these changes. Additionally, we suggest that this insight may be useful for the design of crystalline high-entropy alloys, for which a lower difference in radii is desirable.

Leveraging this insight and the predictive ability of the ensemble neural-network model, we make a number of suggestions for novel binary and ternary glass-forming alloy systems that will exhibit increases in the deviation of atomic radii due to the balancing of electron density. In the search for these alloy systems, lithium frequently appears due its combination of small radius and low *n*_WS_, and has been seen elsewhere in the literature as an addition to alloy compositions which increases GFA.

We emphasize the need for more data to be published on glass-forming alloy compositions, both detailing the successful creation of glassy alloys but, importantly, also failed attempts. This is essential to avoid the study of BMGs becoming entrenched in well-trodden areas of composition-space, dependent on empirical rules. Further, the relative lack of BMG-forming alloy compositions with large *D*_max_ presents a significant challenge to ML, as little information is provided from which to learn the rules that determine their existence. Nevertheless, the transferability of our model is successfully tested *via k*-folds cross-validation.

Future work in this direction may consider more advanced theories than the Thomas–Fermi model for electron density, such as the Thomas–Fermi–Dirac model which includes the exchange energy, or a full density-functional theory treatment. In addition to *n*_WS_, we identify several other features to be important to the neural-network model, of which deeper investigation may return further useful insights into glass formation.

## Data availability

The code written during this work to process data, create neural-network models, and perform model evaluation is available at https://github.com/Robert-Forrest/GFA.

## Author contributions

Robert M. Forrest: conceptualization, data curation, investigation, methodology, software, writing – original draft, writing – review & editing. A. Lindsay Greer: funding acquisition, supervision, writing – review & editing.

## Conflicts of interest

There are no conflicts to declare.

## Supplementary Material

DD-001-D2DD00026A-s001
