# Supplementary material for: Machine-learning improves understanding of glass formation in metallic systems
Source: Digit Discov. 2022 Jun 14;1(4):476–89. doi: 10.1039/d2dd00026a (PMC9358760; doi:10.1039/d2dd00026a)
Supplement: DD-001-D2DD00026A-s001 [file DD-001-D2DD00026A-s001.pdf]

Electronic Supplementary Material (ESI) for Digital Discovery.
This journal is © The Royal Society of Chemistry 2022

Supplementary Material

Machine-learning improves understanding of glass formation in metallic systems

Robert M. Forrest, A. Lindsay Greer

S1 Neural-network background

Neural networks are models constructed of interconnected neuron layers, in analogy to brain architecture.[S1] Neurons receive inputs x and generate an output y, which is directed to connected neurons in another layer.[S2] Each layer may have many neurons, and layers between the input and output layers are *hidden* layers. The *activation*, a, of a neuron is a weighted sum of all the inputs,

$$a = \sum_i w_i x_i \quad , \tag{S1}$$

and the output, or *activity* of the neuron is determined by an *activation function*, of which there are many popular examples, including the *rectified linear unit* (ReLU) function,[S3]

$$\Phi(a) = \max(0, a) \quad . \tag{S2}$$

A network may be described via three interactions, *input to hidden*, *hidden to hidden*, and *hidden to output*,[S4]

$$\begin{aligned} h_1 &= \Phi(W_1^T x) \quad , \\ h_{p+1} &= \Phi(W_{p+1}^T h_p) \quad \forall p \in \{1, \ldots, k-1\} \quad , \\ h_{\text{out}} &= \Phi(W_{k+1}^T h_k) \quad , \end{aligned}$$

where there is a single input layer h_1 and output layer h_{out}, k hidden layers h_p, and W_n is the matrix of weights for a particular layer.

A *deep* NN has many hidden layers, which may be superior to a single hidden layer.[S5] From the universal approximation theory, any smooth function can be approximated by a network with any number of layers, given enough neurons and appropriate weights.[S6] For every smooth function, it is known that there exist NNs that can model it; the task of ML algorithms that train NNs is one of global optimization.[S7]

S2 Full set of candidate features

Table S1 Properties of elements used to calculate linear mixture and deviation features for alloy compositions in the dataset.

Atomic number	Periodic number[S8]	Universal sequence number[S9]
Pettifor-Mendeleev number[S10]	Modified Mendeleev number[S11]	Preferred crystal structure
Atomic radius	Atomic volume	Atomic mass
Electrons	Protons	Neutrons
Group	Period	Series
Block	Debye temperature	Cohesive energy
Electron affinity	Wigner-Seitz boundary electron-density[S12]	First ionization energy
Valence	Valence electrons	s-valence
p-valence	d-valence	f-valence
Pauling electronegativity	Mulliken electronegativity	Miedema ϕ[S12]
Melting temperature	Boiling temperature	Work function
Fusion enthalpy	Vaporisation enthalpy	Molar heat capacity
Thermal conductivity	Thermal expansion	Density
Chemical hardness	Chemical potential	Chemical scale
Shell / valence electrons	Shell / Mendeleev number	

Table S2 Advanced features that are not simple linear mixtures or deviations of individual properties of elements, instead being determined via a specific equation calculated for alloy compositions in the dataset.

Ideal entropy[S13]	$\Delta S_{\text{ideal}} = -\sum c_i \ln c_i$
Xia's ideal entropy[S14]	$\Delta S_{\text{Xia}} = -\sum c_i \ln \frac{c_i r_i^3}{\sum_j c_j r_j^3}$
Mismatch entropy[S15]	$\Delta S_{\text{mismatch}} = \frac{3}{2}\left(\zeta^2 - 1\right) y_1 + \frac{3}{2}\left(\zeta - 1\right)^2 y_2 - \left\{\frac{1}{2}\left(\zeta - 1\right)\left(\zeta - 3\right) + \ln \zeta\right\}\left(1 - y_3\right)$
Mixing entropy	$\Delta S_{\text{mix}} = \Delta S_{\text{ideal}} + \Delta S_{\text{mismatch}}$
Mixing enthalpy[S15]	$\Delta H_{\text{mix}} = \sum_{i \neq j} \Omega_{ij} c_i c_j$
Mixing Gibbs free energy	$\Delta G_{\text{mix}} = \Delta H_{\text{mix}} - T_m \Delta S_{\text{mix}}$
Viscosity[S16,S17]	$\eta = \frac{h N_A}{\sum c_i V_i} exp\left(\frac{\sum c_i G_i - 0.155 \Delta H_{\text{mix}}}{R T_m}\right)$
Theoretical density[S18]	$\rho_{th} = \left(\sum \frac{c_i m_i}{M \rho_i}\right)^{-1}$
Lattice distortion[S19]	$d = \sum_{j \geq i} \frac{c_i c_j \lvert r_i + r_j - 2\bar{r} \rvert}{2\bar{r}}$
Mixing P_{HS} [S20]	$P_{\text{HS}} = \Delta H_{\text{mix}} \Delta S_{\text{mismatch}}$
Mixing P_{HSS} [S20]	$P_{\text{HSS}} = \Delta H_{\text{mix}} \Delta S_{\text{ideal}} \Delta S_{\text{mismatch}}$

S3 Definitions of classification metrics

The metrics used to describe the classification capability of models are defined as follows, where P is the total number of *positives*, N *negatives*, TP *true positives* (correctly predicted positives), TN *true negatives*, FP *false positives* (negatives incorrectly predicted to be positives), and FN *false* negatives:

- *Accuracy*, the percentage of predictions that are correct:[S21]

$$Acc = \frac{TP + TN}{P + N} \tag{S3}$$

- *Recall*, the percentage of positives that are correctly predicted to be positive:[S21]

$$Rec = \frac{TP}{P} \tag{S4}$$

- *Precision*, the percentage of predicted positives that are true positives:[S21]

$$Prec = \frac{TP}{TP + FP} \tag{S5}$$

- *Specificity*, the percentage of negatives that are correctly predicted to be negative:[S21]

$$Spec = \frac{TN}{N} \tag{S6}$$

- F_1 *score*, the harmonic mean of precision and recall:[S21]

$$F_1 = 2\frac{Prec \cdot Rec}{Prec + Rec} \tag{S7}$$

- *Informedness*, the probability that a prediction is being made based on knowledge rather than random guessing:[S21]

$$Inf = Rec + Spec - 1 \tag{S8}$$

- *Markedness*, the probability that the information used by the model is causally linked to the predictions, rather than not being correlated at all:[S22]

$$Mark = Prec + \frac{TN}{TN + FN} - 1 \tag{S9}$$

- *Matthews correlation coefficient*, a measure of correlation between truth and prediction:[S23]

$$MCC = \frac{TP \cdot TN - FP \cdot FN}{\sqrt{(TP + FP)(TP + FN)(TN + FP)(TN + FN)}} \tag{S10}$$

References

S1 V. L. Deringer, M. A. Caro and G. Csányi, Machine learning interatomic potentials as emerging tools for materials science, *Adv. Mater.*, 2019, **31**, 1–16.

S2 A. M. Andrew, *Information Theory, Inference, and Learning Algorithms*, Cambridge University Press, Cambridge, 2003.

S3 V. Nair and G. E. Hinton, *Rectified Linear Units Improve Restricted Boltzmann Machines*, Proceedings of the 27th International Conference on Machine Learning, Haifa, 2010, pp. 807–814.

S4 C. C. Aggarwal, *Neural Networks and Deep Learning*, Springer International Publishing, Cham, 2018.

S5 I. Goodfellow, Y. Bengio and A. Courville, *Deep Learning*, MIT Press, Cambridge, 2016.

S6 G. Cybenko, Approximation by superpositions of a sigmoidal function, *Math. Control Signals Syst.*, 1989, **2**, 303–314.

S7 M. Kubat, *An Introduction to Machine Learning*, Springer International Publishing, Cham, 2017, pp. 1–348.

S8 P. Villars, J. Daams, Y. Shikata, K. Rajan and S. Iwata, A new appoach to describe elemental-property parameters, *Chem. Met. Alloys*, 2008, **1**, 1–23.

S9 Z. Allahyari and A. R. Oganov, Nonempirical Definition of the Mendeleev Numbers: Organizing the Chemical Space, *J. Phys. Chem. C*, 2020, **124**, 23867–23878.

S10 D. G. Pettifor, The structures of binary compounds. I. Phenomenological structure maps, *J. Phys. C: Solid State Phys.*, 1986, **19**, 285–313.

S11 H. Glawe, A. Sanna, E. K. U. Gross and M. A. L. Marques, The optimal one dimensional periodic table: a modified Pettifor chemical scale from data mining, *New J. Phys.*, 2016, **18**, 093011.

S12 A. R. Miedema, A simple model for alloys, *Philips Tech. Rev*, 1973, **33**, 149–160.

S13 B. Deng and Y. Zhang, Critical feature space for predicting the glass forming ability of metallic alloys revealed by machine learning, *Chem. Phys.*, 2020, **538**, 110898.

S14 M. Xia, S. Zhang, C. Ma and J. Li, Evaluation of glass-forming ability for metallic glasses based on order-disorder competition, *Appl. Phys. Lett.*, 2006, **89**, 091917.

S15 A. Takeuchi and A. Inoue, Calculations of mixing enthalpy and mismatch entropy for ternary amorphous alloys, *Mater. Trans., JIM*, 2000, **41**, 1372–1378.

S16 L. Battezzati and A. L. Greer, The viscosity of liquid metals and alloys, *Acta Metall.*, 1989, **37**, 1791–1802.

S17 M. Samavatian, R. Gholamipour and V. Samavatian, Discovery of novel quaternary bulk metallic glasses using a developed correlation-based neural network approach, *Comput. Mater. Sci.*, 2021, **186**, 110025.

S18 K. Bharath, J. A. Chelvane, M. K. Kumawat, T. Nandy and B. Majumdar, Theoretical prediction and experimental evaluation of glass forming ability, density and equilibrium point of Ta based bulk metallic glass alloys, *J. Non-Cryst. Solids*, 2019, **512**, 174–183.

S19 Z. Wang, W. Qiu, Y. Yang and C. T. Liu, Atomic-size and lattice-distortion effects in newly developed high-entropy alloys with multiple principal elements, *Intermetallics*, 2015, **64**, 63–69.

S20 B. Ramakrishna Rao, M. Srinivas, A. K. Shah, A. S. Gandhi and B. S. Murty, A new thermodynamic parameter to predict glass forming ability in iron based multi-component systems containing zirconium, *Intermetallics*, 2013, **35**, 73–81.

S21 D. M. W. Powers, *Recall and Precision versus the Bookmaker*, Proceedings of the International Conference on Cognitive Science, 2003, pp. 529–534.

S22 D. M. W. Powers, *Evaluation: from precision, recall and F-measure to ROC, informedness, markedness and correlation*, Flinders University Technical Report SIE-07-001, 2007.

S23 B. Matthews, Comparison of the predicted and observed secondary structure of T4 phage lysozyme, *Biochim. Biophys. Acta, Protein Struct.*, 1975, **405**, 442–451.
